# Diversity in the Adult and Pediatric Heart Transplant Surgeon Workforce between 2000 and 2020

**DOI:** 10.3390/healthcare10040611

**Published:** 2022-03-25

**Authors:** Olivia Agata Walkowiak, William A. Hardy, Lauren V. Huckaby, Minoo N. Kavarana, Suyog A. Mokashi, Taufiek Konrad Rajab

**Affiliations:** 1 Section of Pediatric Cardiothoracic Surgery, Medical University of South Carolina, Charleston, SC 29425, USA; walkowia@musc.edu (O.A.W.); hardywi@musc.edu (W.A.H.); kavarana@musc.edu (M.N.K.); 2 Department of Surgery, University of Pittsburgh, Pittsburgh, PA 15260, USA; huckabylv@upmc.edu; 3 Division of Cardiac Surgery, University of Tennessee, Memphis, TN 37996, USA; suyog.mokashi@gmail.com

**Keywords:** heart transplant, transplant surgery, workforce, diversity

## Abstract

There is a paucity of literature evaluating trends in the demographic composition of the cardiothoracic surgery workforce. Using the United Network for Organ Sharing database, we retrospectively analyzed the changes in sex, race, and ethnicity of surgeons performing heart transplantations between 2000–2020. Surgeons performing heart transplantations for adult (≥18 years) and pediatric (<18 years) patients between 2000–2020 were identified and stratified by sex (male, female) and by race/ethnicity (non-Hispanic White, non-Hispanic Black, non-Hispanic Asian, Hispanic of any race). Between 2000–2020, the proportion of non-White and female cardiothoracic surgeons performing adult and pediatric heart transplantations increased. Nevertheless, there remains a lack of diversity in the workforce, particularly when compared to the general United States population.

## 1. Introduction

Historically, there has been a lack of female and non-White cardiothoracic surgeons. Several factors within surgical training such as racial discrimination, sexual discrimination, and harassment have been implicated in perpetuating this lack of diversity [1,2]. Despite these factors, recent studies have demonstrated an increase in the number of female cardiothoracic surgeons [3]; however, there is a paucity of literature regarding trends in the racial composition of the cardiothoracic surgeon workforce.

Furthermore, it has been demonstrated that patients undergoing cardiothoracic operations experience sex and racial disparities in care and outcomes [4]. Similar patterns have also been observed among adult patients undergoing heart transplantation (HTx) [5]. Increasing the diversity of the cardiothoracic surgery workforce is one potential approach towards mitigating these disparities. The findings of Greenwood and colleagues have supported this notion by demonstrating that physician–patient racial concordance was associated with improved infant survival after childbirth, which has demonstrable racial disparities in patient outcomes [6]. In a similar study, Greenwood and colleagues found that female patients with acute myocardial infarction treated by female physicians had better outcomes than when treated by male physicians [7]. Moreover, Wallis et al. recently demonstrated that surgeon–patient sex discordance adversely affected outcomes of common surgical procedures [8]. Here, we sought to explore trends in sex and race/ethnicity of surgeons performing adult (≥18 years) and pediatric (<18 years) HTx between 2000–2020.

## 2. Materials and Methods

The United Network for Organ Sharing (UNOS) database was analyzed to explore sex and race/ethnicity among surgeons performing adult (≥18 years) and pediatric (<18 years) HTx between 2000–2020. The study was conducted in accordance with the Declaration of Helsinki, and the Institutional Review Board at the Medical University of South Carolina approved the study protocol and publication of data (Pro00104125). The names of the surgeons performing HTx were identified using the UNOS database, and photographs of these surgeons were obtained through their affiliated institutional websites. Using these photographs, two independent raters classified each surgeon based on sex and race/ethnicity. The classification of subjects into demographic categories by a set of observers is a methodology commonly used in studies in which self-identified demographic information is otherwise unavailable [6,7]. Sex was classified into male or female, while race/ethnicity was categorized into non-Hispanic White (White), non-Hispanic Black (Black), non-Hispanic Asian (Asian), and Hispanic of any race (Hispanic). These classifications were made in accordance with the guidelines on race and ethnicity utilized by the U.S. Census Bureau [9,10]. No other races or ethnicities were considered during this analysis, as an observer methodology was used. Discordance in the identification of sex or race/ethnicity was resolved with the use of a third rater. Concomitant heart–lung transplantations and HTx in which the surgeon’s name was unavailable were excluded. Surgeons without publicly available pictures were excluded from the analysis of surgeon race/ethnicity. If the surgeon’s institutional website identified the surgeon’s sex, surgeons without a publicly available picture were classified by that sex and were included in the analysis of surgeon sex. Descriptive analyses (numbers and percentages) were utilized to present the data. Representation indices were calculated according to the formula *f_surgeons_*/*f_Census_*, where *f_surgeons_* represents the fraction of surgeons corresponding to a particular group, and *f_Census_* represents the fraction of the US population corresponding to the same group.

## 3. Results

### 3.1. Demographics of Surgeons Performing Adult Heart Transplantation

Between 2000–2020, a total of 44,577 adult HTxs were identified through the UNOS Database and included in the analysis of adult HTx surgeon race and ethnicity. Of these, 73.1% were performed by White surgeons, 1.8% by Black surgeons, 5.0% by Hispanic surgeons, and 20.0% by Asian surgeons. In 2000, of all surgeons who performed at least one adult HTx, 88.8% were White, 0.7% were Black, 2.3% were Hispanic, and 8.2% were Asian (Table 1). In 2020, these percentages were 70.8% White, 2.0% Black, 4.4% Hispanic, and 22.8% Asian.

Between 2000–2020, a total of 44,815 adult HTxs were performed, where the surgeon’s sex could be identified. Female surgeons performed 3.0% of these. In 2000, of all surgeons who performed at least one adult HTx, 2.9% were female. In 2020, this percentage became 5.7% female.

### 3.2. Demographics of Surgeons Performing Pediatric Heart Transplantation

A total of 7602 pediatric HTxs between 2000–2020 were identified and included in the analysis of pediatric HTx surgeon race and ethnicity. Of these, 75.7% were performed by White surgeons, 4.2% by Black surgeons, 2.5% by Hispanic surgeons, and 17.6% by Asian surgeons. In 2000, of all surgeons who performed at least one pediatric HTx, 89.3% were White, 0.0% were Black, 2.4% were Hispanic, and 8.2% were Asian (Table 2). In 2020, these percentages were 71.6% White, 4.2% Black, 3.2% Hispanic, and 21.1% Asian.

A total of 7625 pediatric HTxs were performed between 2000–2020, where the surgeon’s sex could be identified. Female surgeons performed 4.6% of these. In 2000, of all surgeons who performed at least one pediatric HTx, 2.4% were female. In 2020, this percentage became 3.2% female.

**Figure 1:**
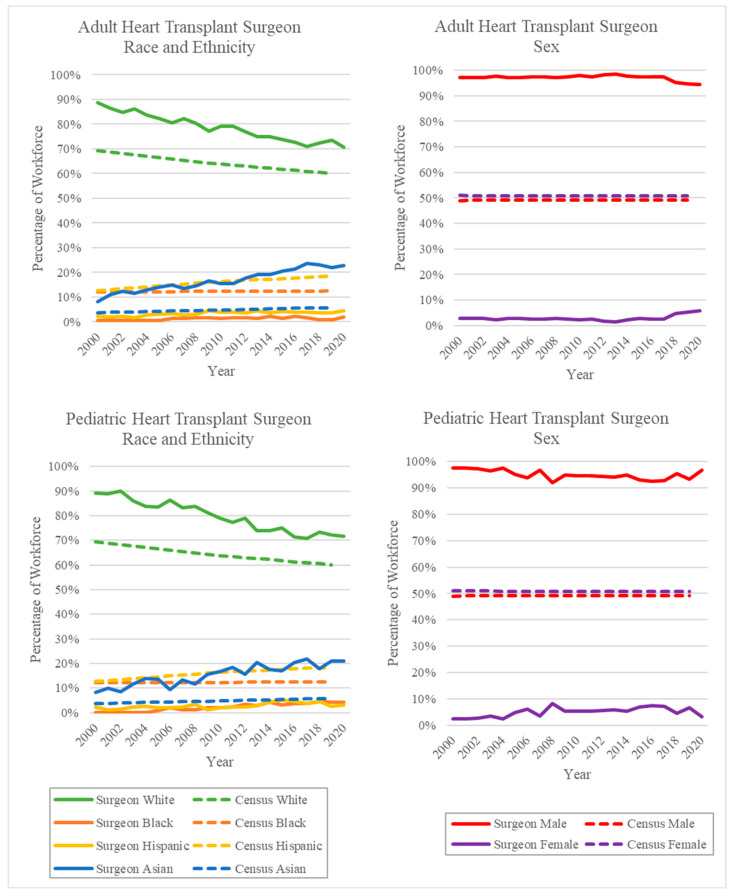
Percentage of workforce by year of surgeons performing at least one HTx. Solid lines represent the HTx surgeon workforce percentage, and dashed lines represent the US population percentages as reported by the US Census [11,12].

**Figure 2:**
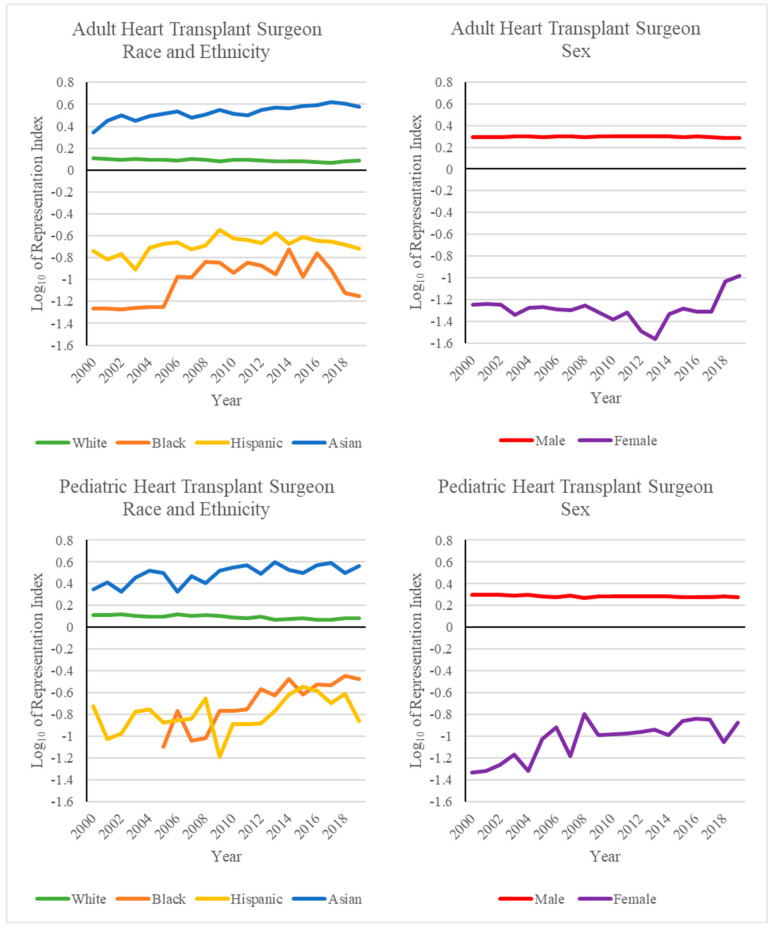
Common logarithm of the representation index by year of surgeons performing at least one HTx. Values greater than zero indicate overrepresentation, and values less than zero indicate underrepresentation. The representation index was calculated with respect to US Census data [11,12].

The adult and pediatric HTx surgeon demographics for each year between 2000–2020 are summarized in Table 1 and Table 2, respectively, and graphed in Figure 1 and Figure 2.

## 4. Discussion

### 4.1. Adult Heart Transplant Surgeon Workforce

The diversity of the adult HTx workforce has increased over the past 20 years. The majority of surgeons in this workforce remain White, although the percentage of White adult HTx surgeons decreased from 88.8% in 2000 to 73.6% in 2019. This roughly mirrors the rate of change of the White population of the United States from 69.4% to 60.1% in the same time period [11,12]. Despite this decrease, the percentage of White adult HTx surgeons was higher than the percentage of the White population of the United States for the entire study period (Figure 1 and Figure 2). The percentage of Asian adult HTx surgeons similarly exceeded the percentage of the Asian population of the US throughout the study period. However, unlike White adult HTx surgeons, the percentage of Asian adult HTx surgeons increased during the study period. Asian adult HTx surgeons comprised 8.2% of the workforce in 2000 and 22.0% in 2019, compared to US Asian population percentages of 3.7% in 2000 and 5.8% in 2019 [11,12]. The percentages of Black and Hispanic adult HTx surgeons remained far below their respective US population percentages during the study period. The percentage of Black adult HTx surgeons increased from 0.7% in 2000 to 2.0% in 2020. The percentage of Hispanic adult HTx surgeons also increased, from 2.3% in 2000 to 4.4% in 2020.

The majority of adult HTx surgeons during the study period were male. Female surgeons comprised 2.9% of the workforce in 2000 and 5.7% in 2020. The percentage of female adult HTx surgeons remained relatively constant from 2000 to 2017 (2.9% and 2.5%, respectively). However, a substantial uptick in the percentage of female adult HTx surgeons occurred between 2017 and 2018, where the percentage of female HTx surgeons increased from 2.5% to 4.7%.

### 4.2. Pediatric Heart Transplant Surgeon Workforce

The diversity of the pediatric HTx workforce similarly increased over the past 20 years. The majority of the pediatric HTx workforce remains White. Despite this, the percentage of White pediatric HTx surgeons decreased from 89.3% in 2000 to 72.3% in 2019. The percentage of White pediatric HTx surgeons remained higher than the White population percentage of the US over the entire study period (Figure 1 and Figure 2). Similarly, the percentage of Asian pediatric HTx surgeons remained higher than the percentage of the Asian population of the US over the same period. The percentage of Asian pediatric HTx surgeons increased from 8.3% in 2000 to 21.0% in 2019. The percentage of Black and Hispanic pediatric HTx surgeons fell far below their respective US population percentages. The percentage of Black pediatric HTx surgeons increased from 0.0% in 2000 to 4.2% in 2020. It is worth noting that prior to 2005 in this study, there were no Black pediatric HTx surgeons. The percentage of Hispanic pediatric HTx surgeons increased from 2.4% in 2000 to 3.2% in 2020.

The majority of pediatric HTx surgeons between 2000 and 2020 were male. The percentage of female HTx surgeons remained relatively constant between 2000 and 2020. The percentage of female HTx surgeons increased from 2.4% in 2000 to 3.2% in 2020.

### 4.3. Limitations

This study utilized raters to classify race, ethnicity, and sex of the surgeons rather than having the surgeons self-identify. Therefore, incongruence may exist between how the raters classified the surgeons and how the surgeons self-identify. The race and ethnicity classifications of this study were limited to four categories (non-Hispanic White, non-Hispanic Black, non-Hispanic Asian, and Hispanic). Other classifications were excluded and were not captured by this study. For this reason, the study was unable to account for multiracial status in addition to immigrant status. Lastly, this study did not investigate gender identity, which is certainly relevant to discussions of diversity.

### 4.4. Trends in Diversity

When looking at the distribution of HTxs by race and ethnicity, it was found that Black and Hispanic surgeons have remained far below their respective US population percentages in the context of both adult and pediatric HTxs. This trend is likely partially due to there being less Black and Hispanic students entering medical school; therefore, there are fewer applicants for positions as HTx surgeons [13]. The proportion of Asian surgeons, on the other hand, has remained above the respective proportion of Asians in the US population. This could be due to cardiac transplants being typically performed at academic centers at which Asian surgical faculty have been previously found to be overrepresented when compared with the US population [14].

The analysis by sex found that the number of female HTx surgeons remained below 5% from 2000–2020. The greatest increase, from 2.5% to 4.7%, was seen from 2017 to 2018 in the context of adult HTxs. This could have been an effect of the steady increase in the number of women in cardiothoracic surgery as a specialty from the year 2000. Additionally, more integrated and fast-track residency programs in cardiothoracic surgery have been created in the past few years [15]. These could be serving as an incentive for women to enter the field, as they allow completion of training in a shorter amount of time. This differs from the discrepancy between the HTx percentages when looking at race and ethnicity, because medical schools do not lack in the representation of women as they do with minority populations. This indicates that there could be a problem during medical school in recruiting women to cardiothoracic surgery, rather than a problem with accepting women to medical school, as is the case with minority individuals.

The general United States population was used as a baseline rather than the practicing physician population because the barriers that exist to becoming a cardiothoracic transplant surgeon also exist for other subspecialties in medicine. Social determinants of health, such as income and access to education, affect the general population from entering the practicing physician population [16]. Structural racism, discrimination, and implicit bias exist as barriers that impact minorities within the medical community [17]. Additionally, a physician workforce should be representative of the population it stems from and serves. The practicing physician population must be explored in its entirety for new trends in diversity before it can be used as a baseline population for a subspecialty of physicians.

This study led to more information about the trends in diversity over the last 20 years within the cardiothoracic surgery workforce in the context of heart transplantation. It was generalized across the entire United States, but it would be interesting to investigate how historical patterns affect the sex and racial disparities across different regions in the United States that are seen today. For example, it could be theorized that there could be a higher proportion of Black surgeons in the Southeastern United States in the current day due to the transatlantic slave trade. Immigration patterns of different races and ethnicities should be explored as well, as minority status is separate from immigration status. The lack of diversity present in the cardiothoracic surgery workforce in the context of heart transplants demonstrates the need to investigate the demographic patterns in other sub-specialized fields of medicine to determine whether this lack of diversity is present across the board, or whether it is only occurring in some fields such as surgery as whole [18]. Future implications of this type of research include investigating the need for policy changes at institutional, community, and societal levels to address this lack of diversity. There should be incentives and support provided for underrepresented individuals at each of these levels of healthcare. These findings serve as objective data illustrating the need for more research to analyze the root of the disparities seen in cardiothoracic surgery.

## 5. Conclusions

Though diversity among cardiothoracic surgeons performing pediatric and adult HTxs has increased over the past 20 years, the majority of the field remains male and White. This is particularly striking when compared with the composition of the United States population (49.2% male, 60.1% White in 2019) [11]. These findings are particularly important, given that prior studies have identified sex and racial disparities in access and outcomes following HTx among adult patients [5], and that racial concordance between physicians and patients improves patient outcomes in settings of known racial disparities [6]. These results demonstrate the need for further research to analyze the causes of sex and racial disparities and initiate more effective efforts to increase diversity of the workforce.

## Figures and Tables

**Table 1:** Composition of adult heart transplantation (HTx) surgeon workforce from 2000–2020. The surgeons listed performed at least one HTx in the indicated year.

	Race and Ethnicity								Sex			
	White		Black		Hispanic		Asian		Male		Female	
Year	No.	(%)	No.	(%)	No.	(%)	No.	(%)	No.	(%)	No.	(%)
2000	270	88.8	2	0.7	7	2.3	25	8.2	303	97.1	9	2.9
2001	263	86.5	2	0.7	6	2.0	33	10.9	300	97.1	9	2.9
2002	259	84.6	2	0.7	7	2.3	38	12.4	302	97.1	9	2.9
2003	254	86.1	2	0.7	5	1.7	34	11.5	292	97.7	7	2.3
2004	243	83.5	2	0.7	8	2.7	38	13.1	287	97.3	8	2.7
2005	239	82.1	2	0.7	9	3.1	41	14.1	286	97.3	8	2.7
2006	247	80.5	4	1.3	10	3.3	46	15.0	300	97.4	8	2.6
2007	255	82.3	4	1.3	9	2.9	42	13.5	304	97.4	8	2.6
2008	225	80.4	5	1.8	9	3.2	41	14.6	274	97.2	8	2.8
2009	219	77.1	5	1.8	13	4.6	47	16.5	278	97.5	7	2.5
2010	224	79.2	4	1.4	11	3.9	44	15.5	278	97.9	6	2.1
2011	226	79.0	5	1.7	11	3.8	44	15.4	279	97.6	7	2.4
2012	233	76.9	5	1.7	11	3.6	54	17.8	298	98.3	5	1.7
2013	215	74.9	4	1.4	13	4.5	55	19.2	283	98.6	4	1.4
2014	223	74.8	7	2.3	11	3.7	57	19.1	292	97.7	7	2.3
2015	223	73.8	4	1.3	13	4.3	62	20.5	294	97.4	8	2.6
2016	235	72.5	7	2.2	13	4.0	69	21.3	316	97.5	8	2.5
2017	229	70.9	5	1.5	13	4.0	76	23.5	315	97.5	8	2.5
2018	230	72.3	3	0.9	12	3.8	73	23.0	304	95.3	15	4.7
2019	248	73.6	3	0.9	12	3.6	74	22.0	320	94.7	18	5.3
2020	211	70.8	6	2.0	13	4.4	68	22.8	282	94.3	17	5.7

**Table 2:** Composition of pediatric heart transplantation (HTx) surgeon workforce from 2000–2020. The surgeons listed performed at least one HTx in the indicated year.

	Race and Ethnicity								Sex			
	White		Black		Hispanic		Asian		Male		Female	
Year	No.	(%)	No.	(%)	No.	(%)	No.	(%)	No.	(%)	No.	(%)
2000	75	89.3	0	0.0	2	2.4	7	8.3	82	97.6	2	2.4
2001	72	88.9	0	0.0	1	1.2	8	9.9	80	97.6	2	2.4
2002	64	90.1	0	0.0	1	1.4	6	8.5	70	97.2	2	2.8
2003	74	86.0	0	0.0	2	2.3	10	11.6	84	96.6	3	3.4
2004	67	83.8	0	0.0	2	2.5	11	13.8	80	97.6	2	2.4
2005	86	83.5	1	1.0	2	1.9	14	13.6	99	95.2	5	4.8
2006	83	86.5	2	2.1	2	2.1	9	9.4	91	93.8	6	6.2
2007	75	83.3	1	1.1	2	2.2	12	13.3	87	96.7	3	3.3
2008	72	83.7	1	1.2	3	3.5	10	11.6	79	91.9	7	8.1
2009	78	81.3	2	2.1	1	1.0	15	15.6	91	94.8	5	5.2
2010	75	78.9	2	2.1	2	2.1	16	16.8	90	94.7	5	5.3
2011	72	77.4	2	2.2	2	2.2	17	18.3	88	94.6	5	5.4
2012	71	78.9	3	3.3	2	2.2	14	15.6	85	94.4	5	5.6
2013	76	73.8	3	2.9	3	2.9	21	20.4	97	94.2	6	5.8
2014	71	74.0	4	4.2	4	4.2	17	17.7	91	94.8	5	5.2
2015	75	75.0	3	3.0	5	5.0	17	17.0	93	93.0	7	7.0
2016	77	71.3	4	3.7	5	4.6	22	20.4	100	92.6	8	7.4
2017	78	70.9	4	3.6	4	3.6	24	21.8	102	92.7	8	7.3
2018	82	73.2	5	4.5	5	4.5	20	17.9	107	95.5	5	4.5
2019	86	72.3	5	4.2	3	2.5	25	21.0	111	93.3	8	6.7
2020	68	71.6	4	4.2	3	3.2	20	21.1	92	96.8	3	3.2

## Data Availability

Not applicable.
